# The Alumni Banquet

**Published:** 1900-06

**Authors:** 


### THE ALUMNI BANQUET.

THE twenty-fifth annual dinner of the Alumni Association of the Medical Department of the University of Buffalo, was given at the Iroquois Hotel, Friday evening, April 27, 1900. Some account of the banquet was given in the May edition of this journal, but our space then was too limited to give all the details.

Two notable speeches were made on that occasion which are worthy of preservation in the records of the Association, hence we print them now. They are transcribed from the stenographer's notes and have not been revised by the authors; nevertheless, they are eloquent and interesting, and deserve to be read by every alumnus.

To the sentiment, Our Alma Mater, Dr. Charles G. Stockton, of Buffalo, responded as follows:

Mr. Toastmaster and Fellow Alumni: I modestly confess that I am a "professor." If I could profess to you as eloquently as Dr. Potter presides, what I have to say would sink deeply into your memories. However, you remember that the professor of whom he spoke merely professed; now, in the matter of speechmaking I do not go so far as that. This is an occasion, however, which stimulates my sluggish faculties; even a duller man than I would be stimulated on an evening like this, in such a company, at the end of the nineteenth century or beginning of the twentieth, or thereabouts, with the spirit that is flowing in this room—overflowing in some parts of it—with a presiding genius that would put good feeling and good cheer into any company, with distinguished guests to the right and to the left of me, certainly to the right, guests whom I have dreaded to follow, and whom I am afraid to precede.

I wish in the beginning to remind you of the fact that in speaking of the Alma Mater to which we cling with so much pride, we are doing no unnatural thing; of course, it is not an unnatural thing to give honor to such a source, but perhaps we do not realise how great an Alma Mater we have. In order to realise exactly what the university represents in its medical department, it is well to reflect upon the status of medical education in this country today, and to compare it with the status of the institutions which existed fifty years ago when this school first came into being. It is hard to realise that we have had only about a century of medical education in this country and that this school goes back somewhat into the earlier part of the century. It is a fact which we forget, that the University of Buffalo is but one of over 150 medical schools in the country; it is a fact which I think we lose sight of, that these schools have more than 4,000 teachers of medicine; that, as shown by the report of the commissioner of education at Washington, based on certificates of two years ago, that more than 5,000 physicians are graduated yearly; and that the property represented by medical schools at that time aggregated about $11,000,000. And it is a sorrowful fact that the endowment fund which all these schools represent, so far as their medical departments are concerned, including those of the great universities of the east and west, all put together, make a sum of less than $2,000,000.

Now we of the University of Buffalo, without the support of a university in the fullest sense of the word, without the support of a community thoroughly given to educational matters, without the support of individual citizens who feel a strong impulse to advance scientific research, have felt at times a little lonely. But when we come to look over the statistics of these 150 schools and find that there is an endowment fund of only about $2,000,000 for all of them, there is a chance to feel that we are not so far behind the great majority of the rank and file of medical schools after all. If we have not endowments we have something that goes a good deal further. If the school without endowments and without this sympathetic support in the community is able to secure the services of several experts in special chairs that require laboratory work, who give their undivided time to the prosecution of pure science, as these men do; if we are able to procure museums and laboratories, and the finest library connected with a medical institution in this country, if we are able to do these things without the support, we have that which is better than an endowment, which in time will bring endowments, a spirit in connection with our Alma Mater which makes things come; and by-and-by we shall look back with pride to the work which some men have done to hold this school to its present plane.

How has it been accomplished? Twenty-five years ago there were only seven professors connected with the school with a few instructors; when I was graduated there was only ten men in the teaching body, while now there are something like eighty, and there has been a marvelous change in the personnel of the staff and in the methods of teaching. There are a good many of you beginning to see why the University of Buffalo is succeeding, not because of the seven,—though they have done their part in bringing this about, how much, it behooves not one of the seven to say,—but because of the seventy men who, unpaid, are performing the more or less thankless task of devoting their time and best effort to the success of the medical department in spite of lack of sympathy from the community. It is this which guarantees the growth and success of the University of Buffalo, a growth which will bring to these men honor in time; for I believe that no man working there today, without salary or the prospect of it, will have done that work regretfully. It is that spirit which makes me hope for the University, and proud of our Alma Mater.

Across several tables from me there sit fifty young doctors of both sexes, whom I am very happy to congratulate and welcome into the ranks of medical practitioners, for which I would ask of them a certain amount of back pay. I believe they will give it. I am going to ask tonight of these fifty graduates a determination to give back to the university such as their predecessors have given; not only that, but a resolution to be members of the Alumni Association *in fact;* a determination to give encouragement to the Association, and to the Alma Mater. It is easy to find reasons not to be active members to work for common ends. But I put it to you, if it is not better for each of us if we all put our shoulders to the wheel and push in one direction, that greater success shall come to the institution, and greater honor follow its name.

The speech of Dr. Stockton was punctuated frequently by applause and on taking his seat he was congratulated by those around him upon the forceful and eloquent manner in which he presented the claims of the university to the favor of the community, and especially to endowment. Let public-spirited citizens respond to his appeal!

To the sentiment, "The Ohio Idea," Dr. Charles A. L. Reed, of Cincinnati, who had delivered the commencement address, responded as follows:

Having engaged your attention already for a considerable time this evening, I feel that it is rather taking advantage of your kind indulgence to inflict myself upon you again, particularly with so commonplace subject as the "Ohio Idea." I say commonplace because it has become so common, you are so familiar with it that it requires no reiteration at my hands. In rising to respond to this toast, however, I purpose taking advantage of the opportunity to exemplify that idea by a certain modest diffidence always characteristic of the Ohio man. Then I want to mention the fact that the Ohio idea has a certain relationship to ceremonies like this, because we have had established over there coeducation in medicine for many years, as well as individual schools. I have great pleasure in stating to my colleagues of the profession that I enjoy the distinction, for I esteem it such, of having been the means of opening the first medical school in the State of Ohio for coeducation. I may say in addition that I was one of the first to close it for the same idea; but this was done in order to establish broader and better privileges in the incorporation of a school for women under circumstances of endowment.

The Ohio people are a peculiar people; they are people who come nearer typifying the great ultimate American composite than those of any other section of the country. Into that great territory migration occurred many years ago with the result that cavalier and puritan settled side by side; and it followed that sooner or later cavalier and puritan corpuscles went coursing merrily together. In that sense we began to realise the great American composite. These converging elements have produced a population that by virtue of its ancestral relationships is in touch with every section of the American continent; and as a result it is sending people with these comprehensive ideas and sympathies over all these United States to broaden the more or less provincial conceptions of the older states. We have exemplified that by sending you here one of our brightest geniuses to develop your great enterprise—the Pan-American Exposition of 1901.

I feel a little reluctant to follow all these gentlemen who have been Chauncey-Depewing it all the way from the straits of Magellan to Baffin's Bay while we have been earning our bread in the sweat of our brows. I am here reminded that we are endeavoring to carry another Ohio idea into effect, and that is in reference to a very strong university organisation. Rapid changes are taking place in our more or less professional institutions which have existed heretofore, and everything is moving in the direction of strong university organisation with well appointed professional and academic departments, sustaining intimate and reciprocal relationships with each other under a strong central government. Now that Ohio idea is one which I should like to see carried out in other parts of the country. We who have had occasion to study the question very seriously feel the necessity for this step, and it is one of the things I expected to discuss in your alumni meeting this afternoon, but the promotion to be your evening orator deprived me of this privilege. I only drop this little hint and permit the thought to bear such fruit as it may in this fertile soil.

I wish to take advantage of this opportunity to emphasise somewhat remarks that have been made by Mr. Director-General Buchanan. I have long been profoundly convinced that the finest professional opportunities offered in the world today are presented in the Latin-American countries. With American enterprise and push and with the genius for a practical application so characteristic of the American mind, our countrymen who have gone there, if not our strongest men, perhaps the more adventurous, have achieved most conspicuous success. I accept in all sincerity what was stated banteringly, that by virtue of my correspondence with every Latin-American country I shall take great pleasure in endeavoring to give an applicant such credentials as shall render him persona grata to any such republics. In several cities of Mexico and Costa Rica, as well as in Quito, are now to be found American physicians who went there after the Pan-American Medical Congress, six years ago, having found little difficulty in acquiring the desired local status. This very idea of reciprocity which has been mentioned as a very important and practical question, is one which was brought into effective shape in Washington in 1893, when we appointed an international commission which has been, and is today, working in connection with the Montevideo Congress for the purpose of bringing about precisely that *entente cordiale* among the republics of this western hemisphere suggested by the Director-General. Now, Mr. President, it being a fact that there is a very large field down there which is very promising, and also a fact that competition is getting pretty stiff here, I wish you would bring your persuasive powers to bear on getting some of these young men into those promising fields. We are now supporting, and supporting liberally, a numerically larger proportion of doctors than any other country in the world by 50 per cent. The per capita allottment of doctors is greater here than elsewhere by 50 per cent., and our sanitary condition is the best, while our death-rate is the lowest.

I am reminded of the very eloquent and forcible remarks made by the clerical gentleman, the Rev. Mr. Richards, who has just addressed you. I agree with everything he has said, and only allude to his address in order to emphasise a few of his adjectives. When he states that it is possible to establish in our patients a wholesome psychic attitude by calling or admitting to the sick room a clergyman, a sensible, tactful clergyman—those were the adjectives he used—I agree with him absolutely. And I agree that it is right to keep a fool out of there, whether he be a fool preacher or a fool doctor. And I hope my good friend will bring some missionary influence to bear in his profession so that we shall not have to study individuals to find out whether it is proper to admit them. We do not want any patent-medicine-almanac preachers in the sick room, nor those preachers who perpetually adorn the advertising pages of religious newspapers. When we have a wholesome, sound, sensible man, such as is the gentleman who as just addressed you, we feel safe, and find a helper in whom we are glad to extend the right hand of cordiality. I am not a Roman catholic, and do not care to make any invidious distinctions, but from the complete abstinence of the clergy of that denomination from all participation in the iniquity of nostrum endorsing, I have no hesitancy, when my patients ask for a priest, to admit him promptly. Therefore, I hope that influence will be brought to bear to place the whole clerical profession in such standing that we shall not have to make discriminations.

And now, Mr. President, permit me to thank you for the opportunity of again inflicting myself on this long-suffering audience, and to detain it just long enough to say how glad I have been to participate in these exercises.

